# Sensitization and Habituation of Hyper-Excitation to Constant Presentation of Pattern-Glare Stimuli

**DOI:** 10.3390/neurolint16060116

**Published:** 2024-11-21

**Authors:** Thomas Jefferis, Cihan Dogan, Claire E. Miller, Maria Karathanou, Austyn Tempesta, Andrew J. Schofield, Howard Bowman

**Affiliations:** 1 School of Computer Science, University of Birmingham, Edgbaston, Birmingham B15 2TT, UK; 2 School of Psychology, College of Life and Environmental Sciences, University of Birmingham, Edgbaston, Birmingham B15 2TT, UK; 3 School of Psychology, College of Health and Life Sciences, Aston University, Birmingham B4 7ET, UK; 4 Functional Imaging Laboratory, University College London, London WC1N 3AR, UK

**Keywords:** pattern glare, headache, migraine, EEG, ERP, discomfort, cortical hyperexcitability

## Abstract

Background/Objectives: Pattern glare, associated with cortical hyperexcitability, induces visual distortions and discomfort, particularly in individuals susceptible to migraines or epilepsy. While previous research has primarily focused on transient EEG responses to patterned stimuli, this study aims to investigate how continuous presentation of pattern-glare stimuli affects neural adaptation over both fine (seconds) and coarse (entire experiment) temporal scales. Methods: EEG recordings were obtained from 40 healthy participants exposed to horizontal square-wave gratings at three spatial frequencies presented continuously for three seconds each across multiple trials. Participants’ susceptibility to visual stress, headaches, and discomfort was assessed using questionnaires before and during the experiment. The experiment employed a two-by-two design to evaluate habituation (exponentially decreasing response) and sensitisation (exponentially increasing response) effects at two different time granularities. Mass univariate analysis with cluster-based permutation tests was conducted to identify significant brain response changes during the period of constant stimulation, which we call the DC-shift period. Results: Significant effects were observed during the DC-shift period, indicating sustained hyper-excitation to the medium-pattern glare stimulus. In particular, the mean/intercept analysis revealed a consistent positive-going response to the medium stimulus throughout the DC-shift period, suggesting continued neural engagement. Participants reporting higher discomfort exhibited sensitisation at fine temporal granularity and habituation at coarser temporal granularity. These effects were predominantly localised to the right posterior scalp regions. Conclusions: The study demonstrates that individuals sensitive to pattern-glare stimuli exhibit dynamic neural adaptation characterised by short-term sensitisation and long-term habituation. These findings enhance the understanding of cortical hyperexcitability mechanisms and may inform future interventions for visual-stress-related conditions, such as migraines and epilepsy. Further research is needed to explore the underlying neural processes and validate these effects in clinical populations.

## 1. Introduction

Pattern glare is characterised by symptoms of perceptual distortions, discomfort, and visual stress when viewing striped patterns [1]. Since striped patterns rarely occur in nature (particularly at the spatial frequencies that aggravate the brain), it is thought that such stimuli take the brain beyond the processing regime for which it has evolved, and the observed hyper-excitation response to such stimuli reflects this evolutionary aberrance [2,3]. Some individuals are more affected by these patterns, particularly those who suffer from visually-induced epilepsy [4], migraines [5] or visual stress [4,6]. As early as 1935, researchers documented the effects of bright lights or patterns on people who suffer from migraines, finding a small number of individuals who had episodes that could be triggered by these patterns or lights [7].

Thus, pattern glare is a well-established phenomenon with sustained research dating from the 1980s on the effects of striped patterns on the visual system of humans. The pattern glare test was formalised in 2001 to allow practitioners to assess an individual’s susceptibility to pattern glare [8]. The test is designed to elicit visual distortions and discomfort. Individuals report the level of distortion and discomfort on a questionnaire with a set of yes/no questions. For an individual to be clinically diagnosed with pattern glare, they must score in or above the 95th percentile in the test. However, there is no objective measure to assess the level of distortion an individual is experiencing; answers to questionnaires are subjective and susceptible to response bias.

More recently, researchers have shown that visual gratings can induce pattern glare, the brain correlates of which can be detected at the scalp level using MEG & EEG, with patterns close to 3 cycles per degree (c/deg) eliciting the greatest response [9,10]. More recently, there have been studies identifying electrophysiological correlates of hyper-excitation, with [11] finding differences between the responses of migraine sufferers and controls in the time-domain. The migraineurs had an enhanced N2 deflection that could be driven by hyper-excitation. Migraineurs’ sensitivity was corroborated with findings from [12], who found that migraine sufferers saw significantly more illusions when viewing striped patterns and would be more likely to select a coloured filter to aid with visual comfort. In a review of the literature, [9] suggested that repetitive patterns, particularly those similar to high-contrast stripes or chequerboards, were implicated as visual triggers for migraines. Additionally, we have previously provided evidence that susceptibility to headaches predicts the absence of the N1 Event Related Potential (ERP) component, i.e., participants with increased headache susceptibility exhibited a smaller N1 and thus a more positive-going response to the stimulus at the aggravating spatial frequency [13]. However, Tempesta et al. did not consider how the ERP response changes through time, thus neither sensitisation nor habituation effects were identified: patterns of change that are likely to be important in understanding migraine, epilepsy, and visual stress.

### 1.1. Change Through Time

Many studies have found a dysfunctional habituation system in migraine and epilepsy, disorders that, as we have indicated, have been linked to a susceptibility to pattern glare [14,15]. As discussed, the possibility of a dysfunctional habituation system was not explored by [13]; we aim to address this shortcoming by analysing how the ERP response to pattern-glare stimuli changes through both their short-term repetition (sequences of identical stimulus presentations) and across the time-course of the whole experiment.

Importantly, treatments for visually-induced migraine and epilepsy could be informed by studying how the brain habituates to pattern-glare stimuli, where that habituation could be over short or long time frames. Accordingly, our experiment has a two-by-two design, with two types of change through time: habituation (exponentially decreasing response through time) and sensitisation (exponentially increasing response through time), and two granularities of time: fine/short-term (through trains of repeated stimulus presentations) and coarse/long-term (over the course of the entire experiment). We also investigate how these types and granularities of change through time are modulated by participants’ state and trait sensitivities to relevant conditions, such as headache, visual stress, and discomfort induced by viewing pattern-glare stimuli. These three sensitivities will be called factors in this work. (Having three factors in this way does increase our vulnerability to type I errors, a point we return to in the discussion, since they emerged from a factor analysis.) In this paper, our main focus will be on the discomfort factor since it gives us our strongest effects, although this may be because it is largely a state measure, reflecting experience during the experiment, rather than a trait measure, reflecting participants’ (more subjective) view of their long-term susceptibility to headaches, visual hallucinations, etc.

### 1.2. Stationary Period and DC-Shift

The vast majority of human electrophysiology experiments consider the transients associated with the onset of a new stimulus [16]. However, this focus ignores another important aspect of the brain’s processing of stimuli: the stationary or evolving response to a continuously presented stimulus. Consistent with the focus on onset transients, most ERP studies only present the stimulus for a short period of time, e.g., a few hundreds of milliseconds. However, a stimulus that stays on for longer will continue to drive the brain, perhaps particularly a brain susceptible to hyper-excitation. To observe this brain response, we presented stimuli for three seconds. A basic feature we observe is that the electrical response does reach a somewhat stationary state with constant stimulation, but with a baseline shift relative to the pre-stimulus period; see Section 3.1.1. In electrical engineering terms, this new baseline could be considered a direct-current effect; hence, we call this the DC-shift period.

In the context of studying hyper-excitation, this sustained aspect of the brain’s processing can be revealing. In particular, it could indicate the inhibitory mechanisms employed in the brain, since these would be engaged during a period of constant stimulation, enabling electrophysiological correlates of inhibition to be observed [17]. The effects we identify could thus serve as biomarkers of hyper-excitation and visual stress in the DC-shift period of the pattern-glare experiment.

### 1.3. Hyper-Excitation

Our experiments will use three stimuli: gratings at 3 c/deg, the aggravating pattern- glare stimulus, and two control stimuli at spatial frequencies on either side of 3 c/deg. According to their spatial frequency, we will call the aggravating stimulus the Medium, and the two control stimuli, Thin and Thick.

We operationalise hyper-excitation as the electrical response of the brain being “larger” for the Medium stimulus than for the Thin and Thick, strictly to the mean of Thin and Thick, although “larger” here actually means more extreme from zero, whether in a positive or negative direction. This is because the polarity of an EEG signal reflects the orientation of the electrical dipole to the recording electrode, meaning that a response of +X micro-volts is in no sense bigger than a response of -X micro-volts.

### 1.4. Hypotheses

To be specific, we have the following hypotheses:

(1) Hyper-excitation will build up through the course of a period of constant stimulation.
(2) Participants experiencing discomfort to the aggravating stimulus, the Medium, will also exhibit sensitisation, i.e., hyper-excitation will increase across repeating presentation, over a relatively fine temporal period, of a few seconds. This is consistent with findings discussed previously that migraine and epilepsy sufferers do not habituate to the repeated presentation of aggravating stimuli.
(3) For those same participants, who experience discomfort to the Medium stimulus, we also believe that they will exhibit a change in hyper-excitation over the coarser time resolution of the entire experiment. However, the direction of this effect was less clear before collecting the data. We may have also seen sensitisation through the course of the entire experiment because we are continuing to drive the system with an aggravating stimulus. However, we may have observed habituation across this longer time period because even our participants who are sensitive to the Medium stimulus have not been clinically diagnosed with migraine, headache, or epilepsy. Accordingly, their brains may successfully habituate to the Medium stimulus with sufficient repetitions.
(4) Finally, it is also possible that we will observe an interaction between the two different time granularities, which might, for example, show that sensitisation to repeated presentations of the Medium stimulus only obtains at the start of the experiment when those exhibiting discomfort are first subjected to the aggravating stimulus, with this effect waning through the course of the experiment, i.e., their brains habituating.

## 2. Methods

**Figure 1:**
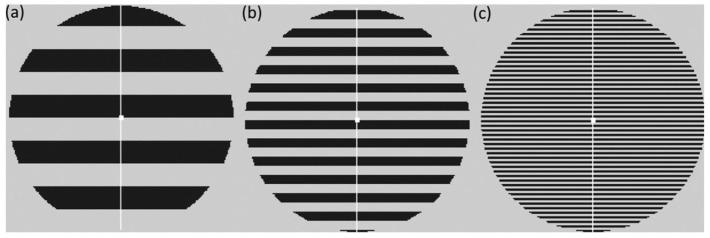
Illustration of the three stimuli used in the pattern glare test and our experiment. (**a**) First control pattern, 0.5 c/deg (thick); (**b**) clinically relevant pattern, 3 c/deg (medium); and (**c**) third control pattern, 12 c/deg (thin). Here the stripes have been scaled so as to avoid distortions in print but are representative of the stimuli shown to the participants.

Stimuli were made with the Psychophysics Toolbox in MATLAB version 2021b (Mathworks, Natick, MA, United States) [18,19,20] and were based on the pattern glare test [8] comprising horizontal square-wave gratings at three different spatial frequencies (SF) (thin = 12 c/deg, medium = 3 c/deg, thick = 0.37 c/deg) with 75% contrast, as illustrated in Figure 1. The stimuli were displayed within a circle with a diameter of 15.2 deg on a grey background matching the mean luminance of the stimuli. They were presented on a Samsung 932BF LCD monitor (Samsung Electronics, Suwon, South Korea) (Resolution = 1280 × 1024 pixels) at a viewing distance of 86 cm. Pattern 1 (thick) is a control stimulus and is not expected to trigger distortions in most participants. It is useful in detecting ‘which patients may be highly suggestible and may respond yes to any question about visual perception distortions’ [1]. Pattern 2 (medium) is the only relevant clinical stimulus falling between SF’s 1–4, which are known to elicit migraines and epileptic seizures [21,22]. Pattern 3 (thin) is a control for poor convergence and accommodation. Those with poor convergence and/or accommodation will see distortions in this stimulus reflecting optical rather than neurological factors [23]. Therefore, for any effect that simply reflects spatial frequency, rather than hyper-excitation, the medium stimulus should have a response close to the mean of the thin and thick stimuli.

### 2.1. Data Collection

Forty participants were recruited at the University of Birmingham, all giving consent by signing the consent form and compensated with £24 for participating. None of the participants had prior history of neurological, psychiatric, or psychological conditions, as well as no history of unconsciousness, convulsions, or epilepsy. Two of the participants were excluded before pre-processing: one withdrew from the experiment before completion, while an equipment failure meant that only a partial dataset was recorded for the other. This study was approved by the Science, Technology, Engineering and Mathematics ethics committee at the University of Birmingham in adherence with the Declaration of Helsinki.

### 2.2. Questionnaires

Multiple questionnaires were used to assess the participants’ headache histories and proneness to suffer from visual stress/pattern glare. These questionnaires included the cortical hyperexcitability index (CHi), used to assess participants’ susceptibility to stimulus-induced hallucinations more generally [24], and the visual discomfort scale (VDS), used for assessing participants’ susceptibility to discomfort in the presence of visual stimuli more specifically [25]. For headache symptoms, we selected questions from a more general headache and general health questionnaire (HGHQ). The headache criteria specified by the International Headache Society [26] were not used, as these are criteria for a clinical diagnosis and do not provide scale measures of headache proneness. The criteria heavily rely on factors such as headache intensity, nature, duration, and frequency. All these factors were recorded by the HGHQ (for more information on the questions used in this questionnaire, see appendix 2 “Headache Questions” in [27], supplementary material).

### 2.3. Procedure

**Figure 2:**
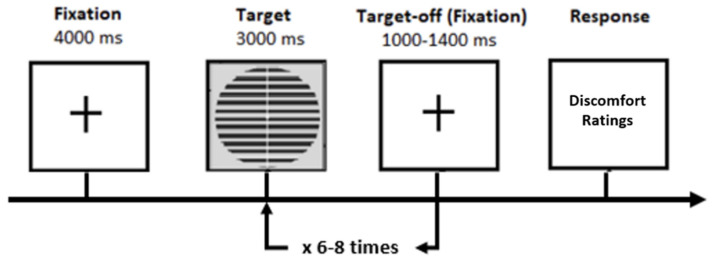
Schematic representation of a single trial. This sequence was repeated 6 times per stimulus type (Thick, Medium or Thin) to complete one block (partition) of experiments.

Following EEG cap setup, there was a five-minute resting period before the main experiment began. The main experiment consisted of three blocks (which we call partitions), with six trials per stimulus type (thin, medium, thick) for a total of eighteen trials per stimulus type across the whole experiment. Every trial began with a fixation cross for four seconds, followed by seven to nine onsets of the same stimulus, each of which stayed on the screen for three seconds. This was followed by another fixation cross for between 1 and 1.4 s; see Figure 2. Following each trial, the participants rated their degree of visual discomfort on a five-point scale (1 = comfortable, 5 = extreme discomfort) and recorded how many times they believed the stimulus was shown to assess their attentiveness. At the end of each block (partition), there was another five-minute resting period where the participant was instructed to rest and close their eyes.

### 2.4. Factor Analysis

Working with the 38 participants who successfully completed the study, we computed the mean discomfort ratings for each participant and stimulus type (thick, medium, thin) across the three blocks (partitions). As discomfort ratings tend to co-vary across the stimulus types, the discomfort index for each participant was computed by subtracting the mean discomfort ratings for the thin and thick stimuli from the scores of the medium stimuli. Thus, we identified participants who found the medium stimulus more uncomfortable than the control stimuli. The overall scores for the CHi and VDS were computed according to their instructions. Lastly, key variables were extracted from the HGHQ, including headache frequency, intensity and duration, and the experience of sensory aura. There was thus a total of seven measures, which had very different ranges, and so were standardised before entering them into the factor analysis. Factor analysis was then used to identify three factors based on a Scree plot analysis. Following a Varimax rotation and an analysis of the factor loadings, these factors were characterised as visual stress, headache, and discomfort. The supplementary material/appendix section ”Factor Analysis” [28] provides a full analysis on the identified factors, which in particular investigates the reliability of our factor analysis, arguing that we can have confidence in the factors we have identified.

This paper focuses on the discomfort factor since it gave us our most substantial effects. We believe this is because the discomfort factor loads more strongly on state measures, as opposed to the other factors that load on trait measures. This suggests that the discomfort factor is tuned to the participants’ response to the stimuli in the experiment in a fashion that the other factors are not. We return to this issue in the Discussion. We focus on effects associated with orthogonalized regressors, although we do also summarise the same effects without orthogonalization in the extended article (see supplementary material/appendix section “Full MUA results” in [28]). In a standard regression, all regressors would be entered together into the regression. Because of limits associated with the Monte-Carlo permutation test provided in Fieldtrip, it is only possible to introduce an intercept and a single non-intercept regressor into each regression model. Consequently, it could be argued to be appropriate to orthogonalize regressors, so that the same variability in the data is not explained by multiple regressors.

The factors were orthogonalized in the order visual stress, headache, and discomfort, using the Gram–Schmidt method (GSM) [29]; this was because visual stress was highest in the Scree plot (highest Eigenvalue), headache was next, and discomfort third.

### 2.5. Data Pre-Processing

The EEG data was first decimated from the recording frequency of 2048 Hz to 512 Hz using the Biosemi toolbox (Biosemi, Amsterdam, Netherlands) and EEGLAB (Swartz Center for Computational Neuroscience, University of California, CA, USA) [30]. Eye blink artifacts were removed using independent component analysis (ICA), and the dataset was then recompiled.

The Fieldtrip toolbox version 20210921 (Donders Institute for Brain, Cognition and Behaviour, Nijmegen, Netherlands) [31] was then used for the rest of the pre-processing stages. The signals were bandpass filtered using an FIR filter with a range of 0.1 to 30 Hz using a Hanning window. The data for each onset were epoched between −200 ms and 4000 ms relative to stimulus onset and referenced to the average of all electrodes.

The DC-shift period was baselined relative to the −200–0 ms time window just before stimulus onset. Our analysis of the DC-shift period seeks to identify stationary as well as non-stationary differences between the three stimuli (thick, medium, thin) during this period. Consequently, it is appropriate to baseline correct to the nearest time period without transients before the DC-shift period, certainly rather than at the start of the DC-shift period itself, where there are significant transients.

Thresholding was then applied with a ±100µV threshold to remove any remaining large artifacts in the data. Participants were then either accepted or rejected based on having a minimum of 20% useable trials for each condition after thresholding, in line with suggestions by Luck [16], leaving us with 34 participants for the basic average of onsets analysis, 32 for the coarse and fine time granularity analysis, and 31 for the three-way analysis (types of analyses are outlined in Section 2.6).

A number of different regressions were performed. These fell into four types, according to the form of the dependent variable employed. In the first type, the dependent variable was the average of the onsets 2–8; onset 1 was excluded from the average as it could contain an effect of surprise (all three stimuli were equally likely at onset one, but the same stimulus was presented for the remaining onsets). Onset 9 was also excluded as it was infrequently shown to participants and was thus considered too noisy. This first type gave us the mean/intercept and Discomfort independent variable regressors discussed in Section 2.6.1. In the second regressor type, the dependent variable was the coarse time granularity, in which the experiment was split into three partitions in line with the experimental blocks, with onsets averaged in each partition. This gave us an idea of how the participants’ brain responses changed throughout the duration of the whole experiment. This second type gave us the coarse change through time-independent variable regressors discussed in Section 2.6.2. In the third regressor type, the dependent variable was the fine time granularity, which compares onsets. Pairs of onsets were averaged together: onsets 2 and 3; onsets 4 and 5; and onsets 6 and 7. Averaging onset- pairs in this way gave us more trials in our analysis bins, increasing the signal-to-noise ratio. These averages were then compared to give us an idea of how the participants’ brain responses changed within each trial. This third type gave us the fine change through time-independent variable regressors discussed in Section 2.6.2. The final regressor type had a similar form to that of the partitions independent variable; however, in this case, each partition was split into three onset-pairs (2:3, 4:5 and 6:7). In this way, we analysed the participants’ progression through both the coarse and fine time granularities. This final type gave us the three-way interaction of our independent variables discussed in Section 2.6.3. We illustrate these regressors more fully shortly.

### 2.6. Mass Univariate Analysis

A mass univariate analysis (MUA) is the analysis of a large number of simultaneously measured dependent variables (e.g., voxels or samples) by performing the same univariate hypothesis tests (e.g., *t*-tests) across all of those dependent variables. This method provides a powerful correction for multiple comparisons. An MUA was conducted on the participants’ ERPs in FieldTrip, using FieldTrip’s cluster-based permutation test method. The significance probabilities of the permutation tests were calculated using the Monte Carlo method, using a significance threshold for FWE-correction of 0.05, with a cluster-forming threshold of 0.025. All tests were run for 10,000 permutations, but then effects with *p* values close to 0.05 (i.e., where passing the 0.05 threshold is uncertain) were run again with 25,000 permutations to obtain a more accurate *p*-value estimate. The MUA was conducted on what we call the Pattern Glare Index (PGI), formed by subtracting the average EEG response for thick and thin stimuli from that for medium stimuli—see Equation (1). This index enables us to focus on parts of the data volume where the medium stimulus exhibited more extreme responses than the average of the two control stimuli. If the brain response reflects changes in purely visual properties, such as spatial frequency, without any relevance to hyper-excitation, the response for medium should sit between the response for thick and thin. Medium being more extreme than the mean of thick and thin suggests hyper-excitation.

(1) PGI=medium−average(thick,thin)

#### 2.6.1. Basic (Non-Temporal) Analyses

**Figure 3:**
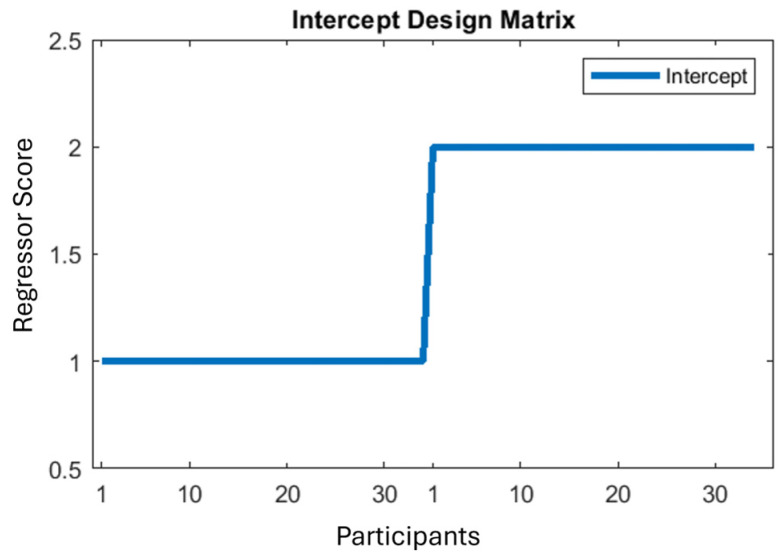
Intercept regressor for the average of onsets 2–8. X axis represents participants, Y axis represents the scores entered in the regressor for each participant. The full set of participants are duplicated on the x-axis. This is because Fieldtrip implements a one-sample *t*-test by performing a 2-sample *t*-test on a duplicated set of participants—see text in brackets beginning ‘Due to the way Fieldtrip’ in previous paragraph.

**Figure 4:**
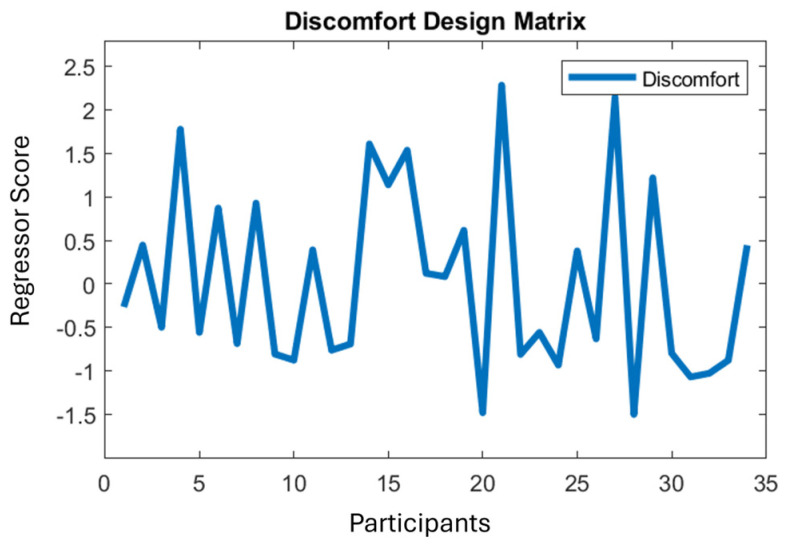
Discomfort regressor for the average of onsets 2–8. X axis represents participants, Y axis represents the scores entered in the regressor for each participant. Thus, a column vector is constructed from the numbers in this panel, which becomes a regressor entered into the regression model. For example, a sample (time-space point) in the EEG data that shows a high response for participants with high values on this regressor and a low response for those low on this regressor will obtain a high coefficient for this regressor, indicating a strong correlation between discomfort score and brain response.

For the first independent variable type, we ran an MUA with a regression with intercept and factor scores; see Figure 3 and Figure 4 (shown for discomfort). (Due to the way Fieldtrip sets up statistical inference for a one-sample *t*-test, it is required to duplicate the data object and to replace all the duplicate’s functional values with zeros. Then this zeroed data is assigned a different integer in the regressor (Figure 3). A two-sample *t*-test is then run over this regressor, which simulates a one-sample *t*-test over a standard (all-ones) intercept regressor.) Since all the factor regressors are mean-centred, the intercept becomes the mean of the basic stimulus effect on the PGI; see Figure 3. This analysis identifies points in the data volume in which medium is more extreme than the mean of the two control stimuli (thin and thick), without considering a participant’s proneness to visual stress, headache or discomfort, or also their sensitivity to change through time. The discomfort regressor shown in Figure 4 is a typical continuous regressor, with a number (*y*-axis) for each participant (*x*-axis), with high numbers indicating a high discomfort response.

#### 2.6.2. Change Through Time

Two additional steps were taken to generate the factors x time granularity regressors; i.e., formulate the interaction between factor and change through time. Firstly, all the factor scores were shifted such that all the scores were positive or zero, this was done by subtracting the minimum from all the scores. These scores were then duplicated three times (one for each time period, i.e., block/partition or onset-pair) and then multiplied by an exponential change pattern, which we explain formally in the extended article’s appendix/supplementary material (see section “Change through time regressors” and Figure 25 of Jefferis et al., 2024 [27]). Lastly, the regressor was mean centred.

**Figure 5:**
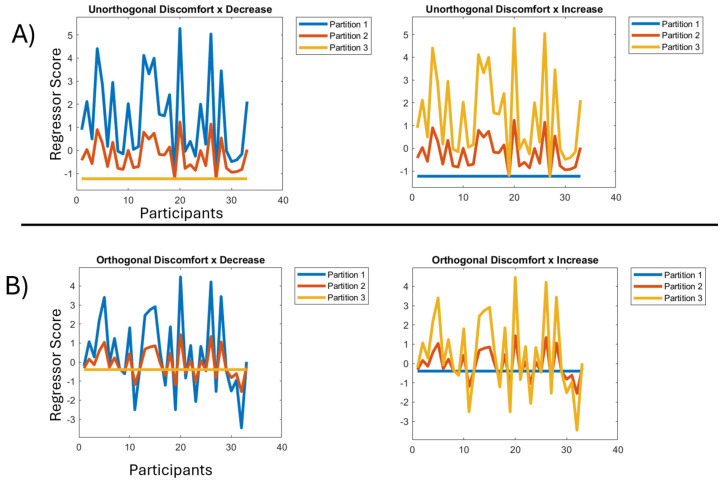
(**A**) Left: regressor for discomfort by decrease across the blocks/partitions before orthogonalization. X axis represents participants, Y axis represents the scores of each participant. Right: regressor for discomfort by increase across the blocks/partitions before orthogonalization. X axis represents participants, Y axis represents the scores of each participant. (**B**) Left: regressor for discomfort by decrease across the blocks/partitions after orthogonalization. X axis represents participants, Y axis represents the scores of each participant. Right: regressor for discomfort by increase across the blocks/partitions after orthogonalization. X axis represents participants, Y axis represents the scores of each participant.

The resulting two-way interaction regressors have one of two forms: (1) a decreasing pattern, characteristic of habituation, through the partitions, and (2) an increasing pattern, characteristic of sensitisation, through the partitions; see Figure 5A. In particular, the exponential change through time periods should be evident. For example, in the decreasing pattern on the left in Figure 5A, the mean across participants in partition 1, i.e., across the blue line, is substantially higher than in partition 2, i.e., across the red line, which is somewhat higher than in partition 3, i.e., across the orange line. Additionally, the extent to which the variability in the discomfort factor is exhibited also varies with partitions. That is, in partition one of the decreasing pattern, we are looking for brain responses that vary very substantially across participants, with those high on the discomfort factor exhibiting substantially higher responses than those low on the factor. Although still present, this variability is substantially reduced in partition 2 and completely absent in partition 3, i.e., by the end of the experiment there is full habituation, with the hyper-excitation (which was differential across the discomfort factor) effectively gone. These factor by decrease and factor by increase interaction regressors will also be used unchanged to explore the pattern of habituation and sensitisation across onset-pairs, our finer granularity of change through time.

The second step was orthogonalizing the three-resulting interaction regressors (one for each factor: visual stress, headache, discomfort), which was again performed using the Gram–Schmidt algorithm; see Figure 5B. The other two interaction regressors were orthogonalized with respect to the Visual Stress by Decrease interaction regressor and themselves, using the Gram–Schmidt process [28]. This sequence of orthogonalizations was chosen because Visual Stress was the factor that obtained the strongest loading in our factor analysis and thus would naturally be preserved unchanged by the orthogonalization.

#### 2.6.3. Three-Way Interaction

**Figure 6:**
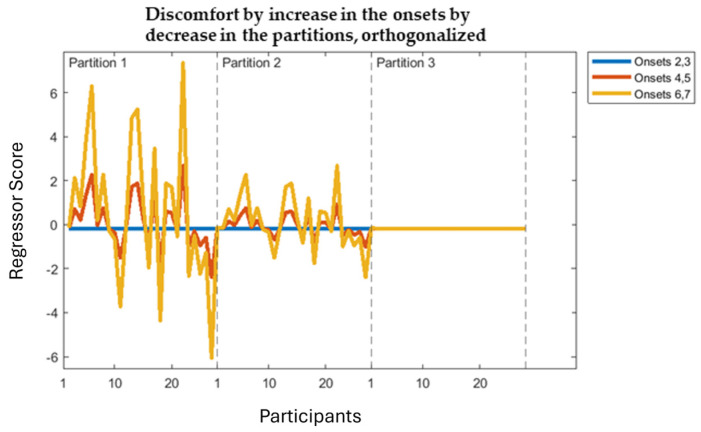
Discomfort by increase in the onsets by decrease in the partitions, orthogonalized regressor. X axis represents participants in each partition with dashed vertical line to mark partition boundaries, Y axis represents the scores of each participant, Onset-pair, Partition that is entered into the regressor.

Each three-way interaction regressor (one for each factor) was constructed by taking the factor regressor from the basic analysis and duplicating this three times (one for each onset-pair); these three were multiplied by the exponential change to form the first change through time effect. Then, these three resulting regressors were appended to form one regressor, which was again duplicated three times (one for each partition). Each of these three (factor by change-through-onset-pairs) regressors was multiplied by the exponential change to form the second change through time effect. The final step of this process was orthogonalizing the three-resulting interaction regressors (one for each factor, visual stress, headache, discomfort), which was again performed using the Gram–Schmidt algorithm. Figure 6 shows an example (orthogonalized) three-way interaction regressor.

### 2.7. Data Visualisation

The MUA provides space-time maps of where in the data volume a regressor statistically significantly explains the data and then returns a space-time map with a mask indicating the identified clusters. This approach does not visualise these significant clusters. Therefore, we created two different visualisations of the significant effects.

**Figure 7:**
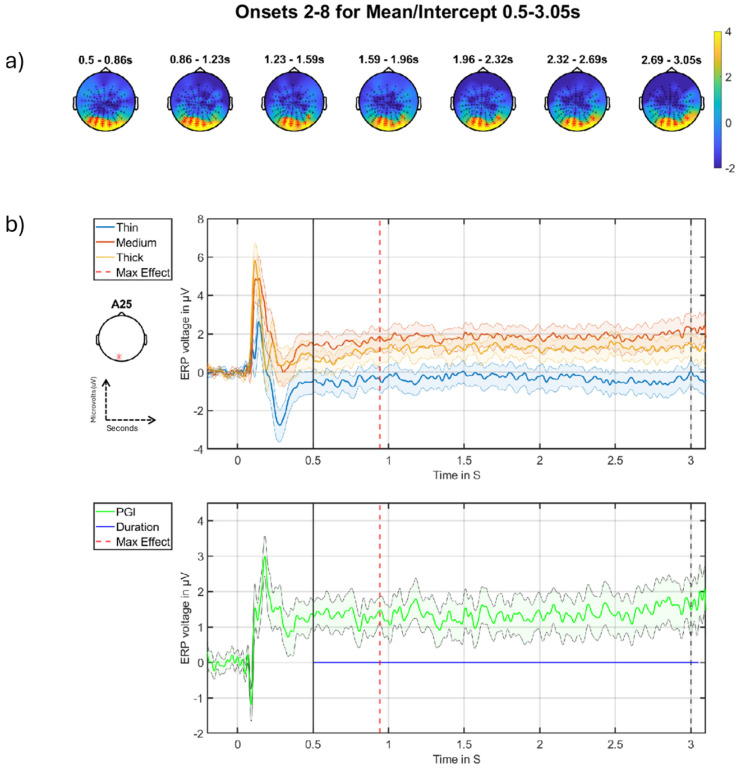
Mean/intercept onsets 2–8 positive cluster in the DC-shift period. (**a**) Topographic maps through time for the whole period, with red crosses indicating the significant cluster, the colour bar on the right represents T-statistic on the topographic maps. (**b**) Grand-averages at electrode A25 as indicated with a red * on left (**b**), with time of maximum effect marked with red vertical line, window start marked with a solid black line, and stimulus offset marked with a dashed black line. Top are the grand-averages for thick, medium and thin, with stimulus onset marked at zero; bottom is the time-series of the PGI, with the blue horizontal line indicating the duration of the effect.

The first is a series of topographic maps, which visualise the location of the most significant cluster on the scalp and how it changes through time, e.g., see Figure 7a. The second visualisation is the grand-average plots, which are created from the electrode in a significant cluster exhibiting the largest effect through time (i.e., with the most timepoints above the significance threshold in the cluster). This electrode is then plotted for the analysis window for each stimulus (thin, medium, thick) and PGI. For factors (not intercept), a median split is performed to visualise the differences between those high and low on a factor.

Grand averages are plotted with bootstrapped 95% confidence intervals (CI). To begin, ERPs from *n* participants are sampled with replacement, where *n* corresponds to the total number of participants. The surrogate grand average of these bootstrapped ERPs is then computed and saved for each condition. This entire process is repeated 5000 times to create a distribution of bootstrapped (surrogate) grand-averages across participants. Finally, confidence intervals around grand-averages are generated by calculating the 2.5% and 97.5% percentiles for each timepoint in the time series based on the generated distribution.

## 3. Results

We show effects during the DC-shift period, which focus on the Discomfort factor, since the vast majority of our effects came out on this factor. The section is split into four subsections: Average of Onsets 2–8 (i.e., mean-intercept), Partitions (coarse time granularity), Onsets 2,3 vs. 4,5 vs. 6,7 (fine time granularity) and the three-way interaction (coarse vs. fine time granularity).

### 3.1. Average of Onsets 2–8

#### 3.1.1. Mean/Intercept Effect

In this section, we present the results for the mean/intercept effect across the collapsed average of onsets 2–8. From Table 1, we can see both the positive and negative tail yield significant clusters in the DC-shift period for the mean/intercept effect. The positive cluster spans the majority of the DC-shift period, whereas the negative cluster only becomes significant towards the end of the window. These positive and negative clusters are likely to reflect the same generator seen from opposite sides of the electrical dipole. The stronger of the two is the positive going cluster, with a *p*-value of 0.0002. This effect is large and spans the entirety of the DC-shift period and is what we will focus on, with the negative cluster being presented in the extended version of this paper (see Figure 11 in [28]).

The most important effect we see is a failure to return to stasis for the medium stimulus (Figure 7b), which is more positive-going throughout the DC-shift period compared to the thick and thin stimuli. This effect is seen across a large portion of the posterior region (Figure 7a), with the maximum effect happening at 0.9414 s after stimulus onset. This suggests sustained hyper-excitation to pattern glare throughout a period of continuous and constant (driving) visual stimulation, which remains, essentially, stable, with some signs of an ongoing increase.

#### 3.1.2. Factors

There were no effects for the Discomfort factor on its own, which is a form of main-effect of our analysis (i.e., not crossed with any other variable). A non-significant effect is reported in the appendix/supplementary material of the extended presentation of these results (see section “Average of Onsets 2–8” in [28] appendix/supplementary material).

### 3.2. Partitions

Since there were no significant effects for the pure change through time effects (i.e., basic increase or decrease effects, which would again be types of main-effect), we now present the discomfort factor effects through the coarse time granularity (partitions) of the experiment (i.e., an interaction of discomfort with time).

#### 3.2.1. Discomfort-by-Decrease Across Partitions

**Figure 8:**
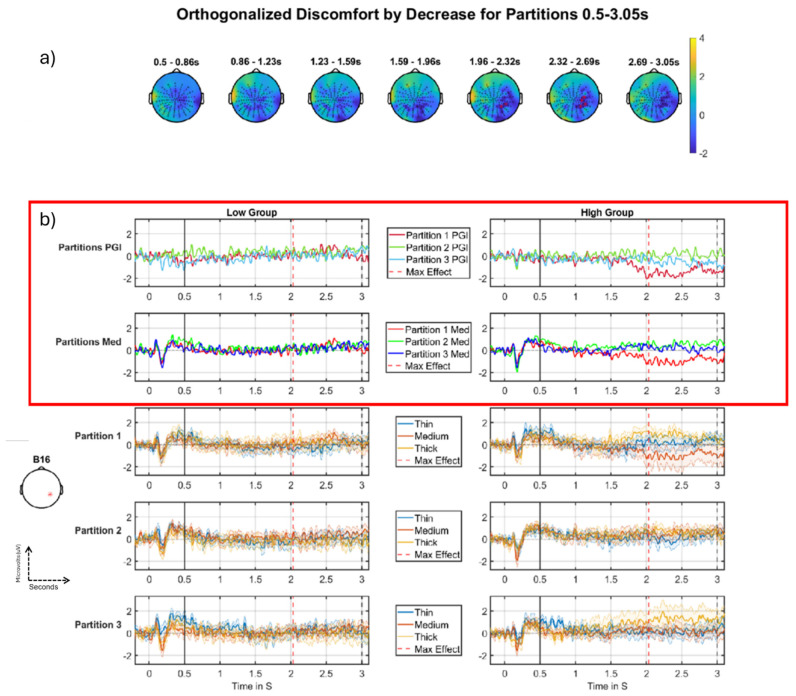
Discomfort- by-decrease across partitions, negative cluster, DC-shift period. (**a**) Topographic maps through time for the whole period, with red crosses indicating the significant cluster, which corresponds to the blue region in (**a**), the colour bar on the right represents T-statistic on the topographic maps. (**b**) Median split (on Discomfort) grand-averages at electrode B16 as indicated with a red * on left (**b**), the left column of grand-averages is for the low group (i.e., low on discomfort factor), right column of grand-averages is for the high group (i.e., high on discomfort factor). Top is the time-series for the PGI for each partition, red (partition 1), green (partition 2), blue (partition 3) with maximum effect marked with a red vertical line, window start marked with a solid black line, and stimulus offset marked with a dashed black line; second row is the grand-averages for the medium stimulus for each partition, red (partition 1), green (partition 2), blue (partition 3); third, fourth and fifth rows present grand-averages for partitions 1, 2 and 3 (respectively), each showing thin, medium and thick. The most important time series comparisons are the PGI and medium stimuli ERPs, indicated with the red outline. The main feature that drives this interaction is a change in the high group from partition 1 to partitions 2/3. This comes out as a negative effect on a decrease across partitions. A negative decrease is an increase, which is what we observe: the response increases from partition 1 (red) to partitions 2/3 (green and blue). Additionally, since this increase is from negative towards zero, we can functionally view this as an habituation effect, i.e., an extreme negative-going response is tending towards what we take as stasis, which here is zero. This pattern is not observed for the low group. That is, the high group on both outlined rows displays the habituation effect, and the low group does not show any clear pattern.

There was a significant negative-going cluster for the discomfort-by-decrease effect across the partitions. The effect lasts over a second and is in the right posterior region of the scalp (Figure 8a), occupying around 10% of the volume at its maximum point (Figure 8a, blue region), with a *p* value of 0.0192 (Table 2, FWE-corrected at the cluster-level). The main feature that drives this interaction is a change in the high group from partition 1 to partitions 2/3 (see top-row, right-side of panel (b). This is a *negative*-going effect on a *decrease* across partitions. A negative decrease represents an effective increase, which is what we observe: the response *increases* from partition 1 (red) to partitions 2/3 (green/blue), i.e., over the course of the experiment. That is, the signal starts negative in Partition 1 and then increases, arriving at a value around zero in subsequent partitions. Accordingly, we view this as an habituation effect, i.e., a progression through time towards the absence of an effect: a PGI of zero (i.e., when there is no difference between Thin, Medium and Thick). In the first two rows in Figure 8b, you can see the high group (right) exhibiting this negative decrease effect; the red line indicates partition 1, green partition 2, and blue partition 3. As just discussed, for the high group, the red line (partition 1) starts as the most negative-going and for the green line (partition 2), the response becomes substantially more positive, arriving at a value around zero. This effect is seen in both the PGI plot (top row) and the medium stimulus plot (second row), suggesting that the effect is driven by a change in the brain response to the clinically-relevant (medium) stimulus rather than to thick and thin. This in turn suggests a reduction (i.e., progression towards a zero baseline) in the hyper-excitation pattern, observed as negative-going at this position on the scalp (anterior to visual cortex). Thus, the hyper-excitation starts around the middle of the DC-shift period and is only present in partition one.

#### 3.2.2. Discomfort-by-Increase Across Partitions

**Figure 9:**
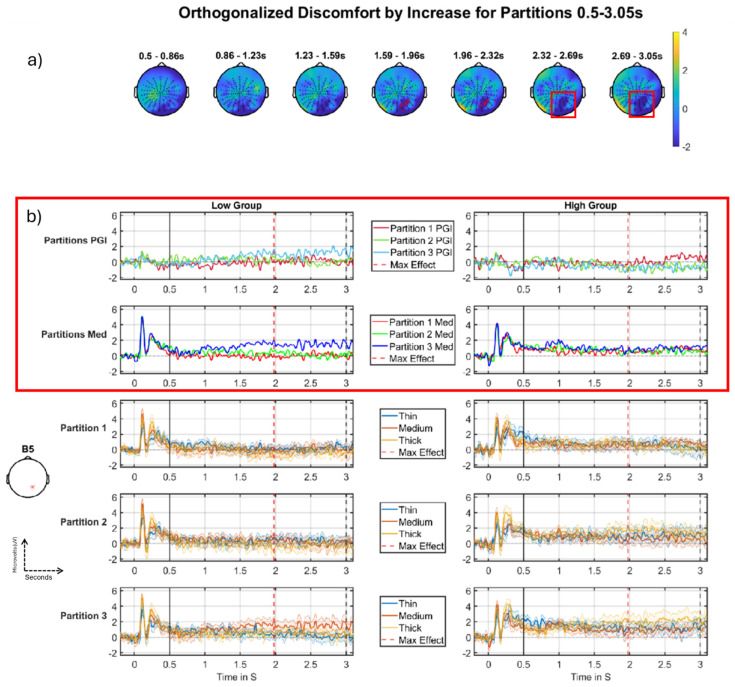
Discomfort- by-increase across the partitions, negative cluster, DC-shift period. (**a**) Topographic map through time for the whole period, with red crosses indicating the first significant cluster the colour bar on the right represents T-statistic on the topographic maps. (**b**) Median split (on Discomfort) grand-averages at electrode B5 as indicated with a red * on left (**b**), the left column of grand-averages is for the low group, right column for the high group. Top is the grand-average for the PGI for each partition, red (partition 1), green (partition 2), blue (partition 3), with maximum effect marked with a red vertical line, window start marked with a solid black line, and stimulus offset marked with a dashed black line; second row is the grand-average for the medium stimulus for each partition, red (partition 1), green (partition 2), blue (partition 3); third, fourth and fifth rows present grand-averages for partitions 1, 2 and 3 (respectively), each showing thin, medium and thick. Although not directly plotted, the second significant cluster is at an adjacent electrode to that plotted (**b**), i.e., A31 is next to B5 and can be seen (**a**) as the last two scalp maps with the red boxes around the clusters’ area. Accordingly, this second cluster is effectively an extension of the one depicted here and can be interpreted from the plots (**b**). The most important time series comparisons are the PGI and medium stimuli ERPs, indicated with the red outline. This makes the effects easy to see, with the low group (left column) on both outlined rows displaying the sensitisation (increasing) effect and the high group (right column) only showing its habituation pattern (decrease) for the PGI.

In the analysis seen in Table 3, there were two significant clusters in a similar location on the scalp with the same direction (tail −1). From the plots in Figure 9, we can say this is likely the same effect that fades briefly and then returns, thus being classified as two clusters. Setting a lower threshold for clustering (or smoothing more) could yield a single stronger cluster. The most significant cluster starts just before 1.5 s after stimulus onset and ends just before 2.5 s (the deep blue region in Figure 9a, topographic maps 4 and 5). The second cluster starts at just before 2.5 s after stimulus onset and ends at 3 s and looks like it is being cut short by the end of the analysis window. The effect we see in the grand-averages (Figure 9b) is seen in the high group (right column of Figure 9b), where we see the PGI becoming more negative (see top row of Figure 9b) as we progress through the partitions. However, there is little evidence of this pattern in the right panel of the second row of Figure 9b, i.e., for the medium stimulus on its own, where all three Partitions sit on top of each other. This in turn suggests that this effect is largely caused by changes across partitions in Thick and Thin rather than in Medium, meaning that this effect is not a particularly clear hyper-excitation effect. Although this is a less compelling effect than the discomfort-by-decrease-across-partitions effect we present in Figure 8, we include it because it plays a role in interpreting the Figure 8 effect in terms of a dipole reversal; see Discussion, subsection “Discomfort habituating through partitions”.

### 3.3. Onsets 2,3 vs. 4,5 vs. 6,7

In this section, we present the results of discomfort through the short time granularity (onsets).

#### Discomfort-by-Decrease

There was a significant cluster for the discomfort-by-decrease effect. At any single timepoint, this cluster is relatively small in size, only reaching a maximum of 5% of the volume; however, it is a strong effect since it lasts over a second with a *p* value of 0.0004 (see Table 4). We ran another analysis where the interaction regressors were not orthogonalized and the results of these are presented as exploratory results in the appendix/supplementary material of the extended paper (see [28], e.g., Figure 27 and Tables 10 and 19).

**Figure 10:**
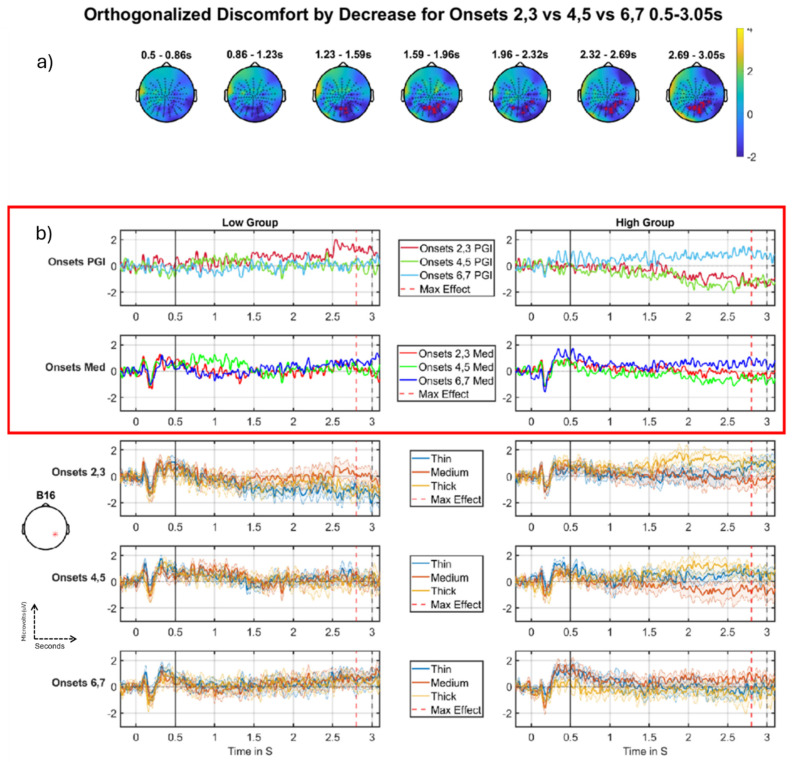
Discomfort- by-decrease across the onsets, negative cluster, DC-shift period. (**a**) Topographic maps through time for the whole period, with red crosses indicating the significant cluster, which corresponds to the blue region in (**a**), the colour bar on the right represents T-statistic on the topographic maps. (**b**) Median split (on Discomfort) grand-averages at electrode B16 indicated with a red * on left (**b**); the left column of grand-averages is for the low group, right column of grand-averages is for the high group. Top are the grand-averages for the PGI for each partition, red (partition 1), green (partition 2), blue (partition 3) with maximum effect marked with a red vertical line, window start marked with a solid black line, and stimulus offset marked with a dashed black line; second row are the grand-averages for the medium stimulus for each partition, red (partition 1), green (partition 2), blue (partition 3); third, fourth and fifth rows present grand-averages for partitions 1, 2 and 3 (respectively), each showing thin, medium and thick. The most important time series comparisons are the PGI and medium stimuli ERPs, indicated with the red outline, The top row (**b**) shows the basic effect, which starts just before 1.5 s. We see a large positive-going change from onset-pair 4,5 to onset-pair 6,7 for the high group, but a negative-going change from onset-pair 2,3 to onset-pair 4,5, for the low group. Additionally, a similar, although weaker, effect can be observed for the medium stimulus for the high group (2nd row (**b**), right hand side), suggesting that the pattern for the high group is not just driven by changes in response for thick and thin, although the low group shows little difference between mediums (2nd row (**b**), left hand side), potentially indicating that the PGI effect in the low group (top row, left side) is driven by changes in thick and thin. That is, the high group on both outlined (see red rectangle) rows displays increasing effect, with the low group almost showing a habituation (decreasing) pattern.

Figure 10 shows the effect, the nature of which is most clearly seen in panel (b). Specifically, the top row of panel (b) shows the basic effect, which starts just before 1.5 s and continues to the end of the segment. We see a large positive-going change from Onset-pair 4,5 to Onset-pair 6,7, for the high group (right side), but a negative-going change from Onset-pair 2,3 to Onset-pair 4,5, for the low group (left side). Additionally, in the same time period, a similar, although weaker, effect can be observed for the medium stimulus for the high group [2nd row of panel (b), right hand side], suggesting that the pattern for the high group is not just driven by changes in the response for thick and thin, although the low group shows little difference between mediums [2nd row of panel (b), left hand side] during the period of the cluster. This suggests that the effect observed for PGI (top row, left side) in the low group is driven by changes in thick and thin, which are less interesting.

Thus, the main phenomenon we observe could be interpreted as an increase in hyper-excitation in the final Onset-pair for those high on Discomfort (light blue line, Figure 10b, right column, top row) and a decrease in hyper-excitation from the first Onset-pair to the second for those low on Discomfort (red line, Figure 10b, left column, top row), although, as just discussed, this latter phenomenon may not be carried by the medium stimulus (Figure 10b, left column, second row), making it less interesting. Notably, although we are looking at the Discomfort-by-*Decrease* interaction, since we are observing a negative effect, the pattern may best be interpreted as Discomfort-by-*Increase*, i.e., an increase through the Onsets for the high group, and, if anything, a decrease through the Onsets for the low group. (The pattern observed in Figure 10b, right panels (high group), is difficult to definitively interpret. One could view the top right panel of Figure 10b as habituation through the onsets from a negative deflection (at early onsets) upwards or sensitization through the onsets towards a positive deflection (at late onsets). We marginally prefer the latter interpretation, because the response to medium (second row), which is more easily interpreted, since it is not calculated across many stimulus types (as the PGI is), ends above zero at Onset-pair 6:7.) Thus, particularly motivated by the pattern for the Medium stimulus in the high group (right side, second row of Figure 10b), we interpret this finding “functionally” as a (differential) sensitisation effect through the onsets for those high on discomfort.

Additionally, these effects seem to be increasing as one approaches the offset of the stimulus, which occurs at three seconds. Thus, it may be that the hyper-excitation is building through the period that the stimulus is on and “driving” the visual system. That is, for those suffering discomfort during the experiment, there is sensitisation at the finest temporal scale, i.e., the three seconds that the stimulus is on for, and also sensitisation at the next temporal scale, i.e., through the sequence of onsets.

Finally, this effect comes out almost as strongly for the equivalent unorthogonalized regressor (see appendix/supplementary material of section “Onsets 2,3 vs. 4,5 vs. 6,7 Unorthogonalized results” in [28]). This indicates that the effect we are observing in this section is not carried by some quirk of the orthogonalization process, giving greater credence to the effect’s reliability.

### 3.4. Three-Way Interaction

#### Discomfort-by-Increase Across the Partitions by Decrease Across the Onsets

**Figure 11:**
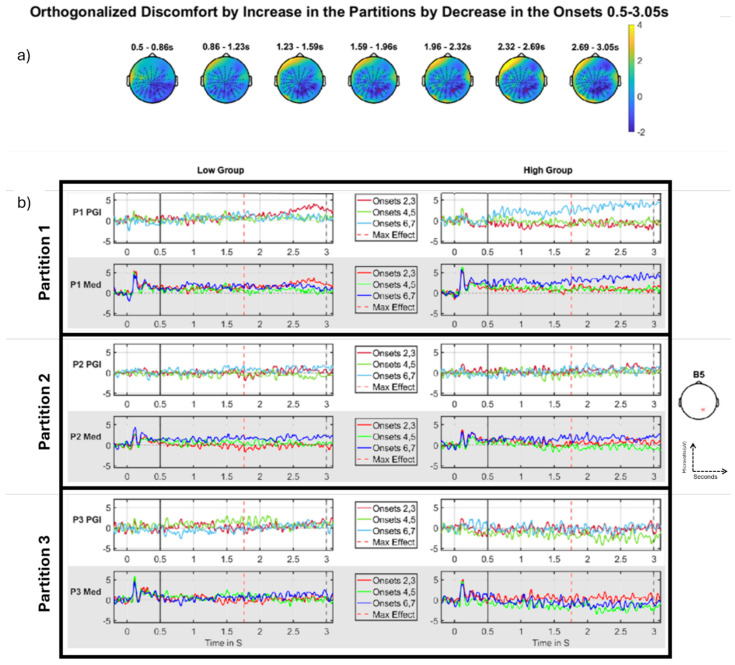
Discomfort- by-increase (through the partitions) by decrease (through the onsets), DC-shift period. (**a**) Topographic maps through time for the whole period, with red crosses indicating the significant cluster, the colour bar on the right represents T-statistic on the topographic maps. (**b**) Median split (on Discomfort) grand-averages at electrode B5 as indicated with a red * on right (**b**); the left panel of grand-averages is for the low group, right panel of grand-averages is for the high group. Top row (partition 1), third row (partition 2) and fifth row (partition 3) show grand-averages for the PGI for each onset, red (onsets 2,3), green (onsets 4,5), blue (onsets 6,7) with maximum effect marked with a red vertical line, window start marked with a solid black line, and stimulus offset marked with a dashed black line. Second row (partition 1), fourth row (partition 2) and sixth row (partition 3) are the grand-averages for just the medium stimulus for each onset, red (onsets 2,3), green (onsets 4,5), blue (onsets 6,7). (**b**) shows what underlies this effect. The first row (partition 1) shows a striking sensitisation effect for the high group (right side), with a substantially higher response in the final Onset-pair (6,7). This pattern is absent, and potentially reversed into a decrease pattern for the corresponding low group grand-averages (left side of first row). Additionally, this increase across Onsets for high and weak decrease for low in partition 1 is also present when we plot the medium alone (2nd row), suggesting the elevated Onset-pair 6,7 effect for the high group truly reflects hyper-excitation. In contrast, the remaining 4 rows (**b**), which correspond to partitions 2 and 3, exhibit no, or certainly much weaker, patterns of change through the onsets. Panels are colored in grey and white only to separate each different panel into different rows.

Although not quite significant (see Table 5), the three-way interaction is qualitatively present in the grand-averages (Figure 11b). Again, this is observed as a negative-going effect, whereby a decrease across the onsets reflects sensitisation and an increase across the partitions, an habituation. The first row (partition 1) shows a striking PGI sensitisation effect for the high group (right side), with a substantially higher response in the final Onset-pair (6,7, light blue line). This pattern is absent and potentially reversed into an habituation pattern for the corresponding low group grand-averages (left side of first row). Additionally, this sensitisation across Onsets for high and weak habituation for low in partition 1 is also present when we plot the medium alone (2nd row), suggesting the elevated Onset-pair 6,7 effect for the high group truly reflects hyper-excitation. In contrast, the remaining 4 rows of panel (b), which correspond to partitions 2 and 3, exhibit no, or certainly much weaker, patterns of change through the onsets.

Thus, the key phenomena are in Partition 1, with the high group showing a clear sensitisation effect, including very substantial hyper-excitation in the final Onset-pair. This suggests that the process of repeating the aggravating stimulus (over a short timeframe of 10 s of seconds) increases the brain’s sensitivity to that stimulus.

When we look through the partitions, we see an habituation effect: the differential sensitisation effect that we observe in partition 1 (sensitisation through onsets for high group and not for low group) reduces from then on, effectively being absent in partitions 2 and 3. Thus, this effect suggests a selective hypersensitivity when those susceptible to pattern-glare stimuli start the experiment, which their brains successfully quench through the course of the experiment. Note this is not the regressor we presented in the Section 2 (see Figure 6), since the three-way that came out exhibited a decrease across onsets and an increase across partitions. However, as previously highlighted, this effect was negative-going, which makes it intuitively like the positive-going pattern of the three-way regressor presented (increase in the onsets and decrease in the partitions). We ran another analysis where the interaction regressors were not orthogonalized; however, no clusters were identified in the MUA and they are thus not presented.

Though not significant, this effect does occur in a region where we have seen the discomfort effects for previous significant clusters. In particular, this three-way interaction is a decomposition of the two-way interaction we observed in Figure 10: it happens at the same position on the scalp, in a similar time interval.

With regards to this effect failing to be significant, it is important to note that adding an extra factor to an interaction (going from two-way to three-way) will reduce statistical power, since the data needs to be distributed across more bins. Additionally, we are performing statistical inference in a large volume (the entire DC-shift period), which reduces the statistical power of the FWE-correction we perform with Fieldtrip. There is also a second cluster identified in the period, which is not close to significant (*p*-value of 0.131). However, this cluster occurs at a similar position on the scalp. This 2nd cluster can be seen as the deep blue region around electrode B5 in the final scalp map of Figure 11a. With more participants or more temporal smoothing, these two clusters could join to become a single large cluster, which would likely be strongly significant.

## 4. Discussion

Through our analysis, we have discovered multiple features in our ERPs that, in our data, correlate with the discomfort factor across several independent variable types, and we have also shown effects for the mean/intercept. There may be more effects in the DC-shift period not uncovered by this analysis. This is because statistical inference using FieldTrip favours effects that span a longer period, sometimes not finding short effects as these are washed out by the familywise error correction performed in the large analysis window. We were unable to split the DC-shift period in an unbiased way to try to find these short effects, as the DC-shift period is smooth and does not have any change of stimulus presentation, which causes sharp changes in the participants’ ERPs, like the ones seen in the onset of the stimulus.

Additionally, it is likely that expectation effects are present in our findings. Specifically, the length of time a stimulus is on for is fixed, and it is highly likely that such predictability is detected by the brains of our participants. Indeed, such expectation effects are commonly observed in event-related potentials, e.g., the contingent negative variation [32]. This does not, though, in any way invalidate our findings, which should be considered within this context. In particular, whether representing predictions or not, the electrical responses we report are all differential across spatial frequencies, exhibiting more extreme responses for the (clinically relevant) medium stimulus. Thus, if one does want to entertain the expectation/prediction interpretation, our findings suggest that expectations are stronger for the medium stimulus, and specifically so for those who report discomfort from viewing this stimulus type (i.e., the high-group). Additionally, since most of our effects seem to build up towards the end of the DC-shift period, the expectation may specifically be for the end of the DC-shift period. That is, it may be that early in the experiment, those susceptible to discomfort are anticipating the aggravating stimulus going off, e.g., see the light blue line on the right in the top row and the darker blue line on the right in the second row of Figure 11b. However, it is also possible that we are simply seeing a growth in hyper-excitation during the DC-shift period driven purely bottom-up from the aggravating stimulus. Further research is required to differentiate these interpretations.

As we have indicated elsewhere, we believe that the findings presented in this paper should be considered as exploratory, requiring replication before they can be considered as robust phenomena. However, it is worth considering our vulnerability to type-I errors, i.e., to false positive findings. We consider this question in the following points.

1. Although we have made a number of comparisons, the number may not be unusual compared to higher-order ANOVAs that are routinely run. Additionally, the regressors we tested, e.g., the two-way interactions, are specifically formulated to test our prior temporal hypotheses, such as a factor interacting with habituation. Indeed, as indicated elsewhere, these two-way interactions are not the “standard” two-ways; they are tailored to our particular hypotheses. Having a strong hypothesis reduces vulnerability to type I errors.
2. Also, we have been very conservative with regard to our analysis window. Specifically, we have undertaken a whole-volume analysis without systematically splitting the full analysis segment into sub windows, or indeed applying any sort of ROI. Additionally, that whole-volume is very large in comparison with what is typical in human-EEG cluster inference, viz. 2.5 s, when one would rarely see an analysis segment beyond 1 s. Such a large volume reduces statistical power with cluster inference; thus, our ability to find significant effects indicates the robustness of our findings.
3. Additionally, some of our *p*-values are very small, e.g., the mean intercept *p*-value is 0.0002, and discomfort-by-decrease for onsets (a sensitisation effect) has a *p*-value of 0.0004. One would have had to perform an enormous number of comparisons before *p*-values of this size would become likely false positives. Although, of course, other of our effects are much weaker and should be viewed with more caution.

### 4.1. Mean/Intercept Effects

The mean/intercept effects correspond to those investigated for the onset transients by [14]. However, here we seek to build on the investigation in that study by looking at the mean/intercept through the major part of the grand-average, i.e., for the DC-shift period.

In the DC-shift period, there is a large effect that is present throughout the entire period; see Figure 7 (between vertical black solid and dashed lines). This effect on the medium stimulus follows a similar pattern to the control stimuli (thick and thin) but at a higher amplitude, suggesting that the medium stimulus continues to drive the brain throughout the DC-shift period.

### 4.2. Effects on Discomfort Factor

Overall, we have found multiple significant effects on the discomfort factor; these effects could help in future diagnosis methods for visual discomfort or cortical hyperexcitability. Although our research is exploratory, it does provide a consistent position on the scalp where the discomfort effects have been present. The effects observed at this position on the scalp could potentially provide a biomarker for the diagnosis of visual discomfort. A possible application of such a biomarker is in drug discovery, where, for example, the effectiveness of a drug to control hyper-excitation could be quantified by assessing its impact on an EEG marker of hyper-excitation. Additionally, source localisation of our discomfort effects could help to understand the neurological aetiology of hyper-excitation, i.e., where and how in the brain hyper-excitation manifests. This could provide a target for electrical or magnetic stimulation, which might be targeted at damping down hyper-excitation. This said, considerable further research will have to be conducted before such applications could be pursued.

Why then did discomfort give us our most substantial effects? We believe this is because the discomfort factor loads more strongly on state measures. That is, we entered into our factor analysis both the results of questionnaires collected at the start of the experiment and discomfort responses collected during the EEG experiment proper. The questionnaires focused on participants’ subjective sense of their sensitivity to relevant symptoms throughout their life, e.g., their sense of how frequently they have headaches. Thus, these are trait sensitivities to relevant symptoms. In contrast, the discomfort state measurements were derived from asking each participant during the EEG experiment whether the stimulus presentation was making them feel unwell/nauseous.

The discomfort factor loaded more strongly on these state measurements of participants feeling unwell/nauseous during the experiment. This suggests that the discomfort factor is tuned to the participants’ response to the stimuli in the experiment in a fashion that the other factors are not. This might tie the discomfort factor more directly to the activation patterns exhibited in participants’ brains in response to the stimuli, and perhaps particularly in response to the Medium stimulus. This may be why discomfort effects are the largest.

#### 4.2.1. Discomfort Habituating Through Partitions

For the partitions, we observed two two-way interactions (see Figure 8 and Figure 9). The first of these (Figure 8), was the more compelling, but the second (Figure 9) was also included for explanatory purposes. Focusing initially on the second of these two-way interactions, the Discomfort-by-Increase analysis in the DC-shift period (see Figure 9) shows an habituation effect for the high group of participants (in the DC-shift period, Figure 9, this comes out as a negative-going effect on an increase interaction, which due to its negative-direction can be interpreted as an habituation effect), although the effect was not present for the Medium stimulus; see Figure 9b, right column, 2nd row. This finding may suggest that those participants who suffer from higher discomfort habituate through the course of the whole experiment, whereas those who report lower discomfort seem to have little or no visible habituation effect (indeed, sometimes a reversed effect).

There is literature to support the hypothesis of habituation in the visual cortex when presented with visual stimuli [33,34]; however, there is conflicting evidence to support this habituation in migraineurs [10,35,36,37], with the majority of the literature suggesting that migraineurs have dysfunctional inhibitory mechanisms leading to no habituation or even sensitisation. Since our study was conducted on a healthy, rather than a clinical, group, our results should be validated on migraine suffers as they may not experience any habituation effect, in line with evidence found in [10,37].

Interestingly, our most compelling two-way interaction on the partitions, Figure 8 and Table 2, also suggest an habituation effect through the partitions that is differential for the discomfort factor. However, this habituation is of a negative-going hyper-excitation pattern in partition 1. Thus, it involves a positive-going change through the partitions, back towards what can be viewed as a zero baseline manifesting as a negative habituation effect. Notably, this high group through the partitions, negative-to-positive pattern in Figure 8, is close on the scalp to the positive-to-negative pattern for Figure 9, with the latter more posterior and closer to the midline. Overall, the Figure 8 and Figure 9 findings seem to indicate that those who suffer from higher discomfort habituate through the course of the entire experiment, whereas those who report lower discomfort do not. This could indicate that those low on the discomfort factor do not experience the same hyper-excitation from the pattern-glare stimuli at the start of the experiment compared to those who are high on the factor, removing the need for the low group to habituate.

Interestingly, the majority of our discomfort effects were observed in right posterior areas on the scalp (around electrode B16), with some of these at somewhat different time points. This may suggest a common electrical source. However, in other work, we have observed discomfort effects very posterior on the left side [27], although those findings were made with a region of interest analysis in which the area around B16 was not included. Accordingly, these two different findings may not be as inconsistent as they seem. More research is required to find the generator of these effects. Currently, there is some literature to support the location of these findings [38]; however, the findings are linked to habituation when viewing complex visual patterns and not discomfort or pattern glare.

#### 4.2.2. Sensitisation Through the Onsets for the Discomfort Factor

We have found results to support the hypothesis of sensitisation through the fine time granularity in the DC-shift period. We see this effect in Figure 10 and Table 4. This contrast was formalised as a negative-going decrease effect for the onsets (2,3 vs. 4,5 vs. 6,7) in which we see onsets 2,3 and 4,5 strongly negative for the high group (see Figure 10b, top row, right panel, green and red lines) and then onset-pair 6,7 jumps to be positive-going (see Figure 10b, top row, right panel, light blue line), which we tentatively (see footnote in the subsection “Discomfort-by-decrease”) interpret as a sensitisation effect. This same pattern is also apparent when looking at the medium stimulus on its own (see Figure 10b, 2nd row, right panel), suggesting that this is a real hyper-excitation effect rather than being carried by thick and thin.

Sensitisation effects through the onsets that are differentially observed across a factor, here discomfort, are potentially of considerable interest since they could represent the electrophysiological correlates of the process by which those sensitive to visual stress become aggravated by visual stimuli. The particular pattern of aggravation we observe in Figure 10b, (see right side, top two rows) may suggest that this aggravation can obtain in two ways: (1) by simply leaving an aggravating stimulus on (the effect is observed towards the end of the stimulus presentation period), and (2) by repeating that stimulus (sensitisation through the onsets).

#### 4.2.3. Three-Way Interaction

To unify the two-way interactions we observed for partitions and onsets, we specifically set-up a three-way interaction. Accordingly, this three-way investigation should be considered particularly exploratory; indeed, this three-way contrast is not orthogonalized with regard to either of the two-way analyses. Thus, the same variability is likely to be driving two- and three-way effects. More specifically, the three-way interaction was setup according to the (statistically) strongest of each of the two-way interactions, increase across the partitions, and decrease across the onsets. This is the opposite of our hypothesis in which we predicted that there would be habituation through the partitions and sensitisation through the onsets. The result for the three-way interaction was not significant (*p* = 0.0686); however, this effect looks consistent with our hypothesis for the change of condition through onsets and partitions as it is negative-going. The grand-averages in Figure 11b show a short-term sensitisation (across the onsets) in the high group (see right side, top and second rows, responses increasing through Onsets) that diminishes over the three partitions (see right side, third, fourth, fifth and sixth rows).

Interestingly, there may be further evidence here of an even finer temporal grain sensitisation for the high group. That is, in Figure 11b, right side, during the final Onset-pair (6,7), there seems to be a progressive increase in PGI (top row, light blue) and the response to the medium stimulus (second row, dark blue line) during the DC-shift time period. This suggests that for those sensitive to the stimulus, continuing to “drive” the visual cortex with a sustained pattern glare stimulus causes the brain’s response (indeed, its level of hyper-excitation) to continue to ramp upwards, with this obtaining over a 3 s period of time.

There may also be habituation during the first partition for those low on the factor. That is, for the group that is not sensitive to discomfort (see left side of Figure 11b), the first Onset-pair (2,3) may be exhibiting a hyper-excitation response late in the DC-shift time period (top two rows, red lines), which dissipates by the second Onset-pair (green line). Thus, it may be that the well-functioning brain is initially aggravated by the pattern-glare stimulus (i.e., for early presentations of the medium stimulus at the start of the experiment), but is then able to habituate to that stimulus extremely rapidly. (The question that remains about this low group pattern is: if it does reflect hyper-excitation, why does that particular pattern, i.e., an early Onset-pair, early in the experiment, exhibiting an elevated response, not obtain for the high group? One might have thought that the high group would exhibit this pattern to a very marked extent.)

## 5. Conclusions

Our findings suggest that participants who reported greater discomfort to the pattern-glare stimulus displayed sensitisation at a fine time granularity, and habituation at a longer granularity. This suggests the presence of sensory impairment, which is consistent with the current literature on cortical hyper-excitability, although the particular pattern that we observed, short-term sensitisation and long-term habituation, is new.

A fundamental question that remains is whether the effects we observe reflect the direct manifestation of hyper-excitation or, alternatively, inhibitory processes initiated in response to that hyper-excitation. Investigations in the frequency domain, where particular frequencies have been associated with inhibition [39], may help to understand this question. Additionally, it needs to be recognised that the research presented here is fundamentally exploratory: we have performed a number of comparisons (see section “Full MUA results” of the appendix/supplementary material in Jefferis et al., 2024 [27]) to identify our main findings (although many of these were correlated), and thus, our effects need to be replicated before they can be considered robust. We also need to see if corresponding effects are also present at other points in the time-course of an ERP segment, i.e., for the onset and offset transients, before and after the DC-shift period.

## Figures and Tables

**Table 1:** MUA results for mean/intercept effect for the average of all onsets. Only results for clusters containing significant effects (FWE-corrected) or borderline effects smaller than a *p*-value of 0.1 are shown, both positive and negative tails.

Effect	Tail (+1)	Tail (−1)
Mean/ intercept 0.5–3.0 s	1st cluster *p*-value: 0.0002 1st electrode: A25 1st peak time: 0.9414 1st r correlation effect size: 0.79 1st Cohens d effect size: 2.59	1st cluster *p*-value: 0.0318 1st electrode: C13 1st peak time: 2.6914 1st r correlation effect size: −0.65 1st Cohens d effect size: −1.69

**Table 2:** MUA results for discomfort-by-decrease through the partitions with orthogonalized regressor. Only results for clusters containing significant effects (FWE-corrected) or borderline effects smaller than a *p*-value of 0.1 are shown, both positive and negative tails.

Effect	Tail (+1)	Tail (−1)
Discomfort by decrease 0.5–3.0 s	No significant Cluster	1st cluster *p*-value: 0.0192 1st electrode: B16 1st peak time: 2.0332 1st r correlation effect size: −0.48 1st Cohens d effect size: −1.1

**Table 3:** MUA results for the discomfort-by-increase through the partitions with orthogonalized regressor. Only results for clusters containing significant effects (FWE-corrected) are shown, both positive and negative tails.

Effect	Tail (+1)	Tail (−1)
Discomfort by increase 0.5–3.0 s	No significant Cluster	1st cluster *p*-value: 0.0108 1st electrode: B5 1st peak time: 1.9766 1st r correlation effect size: −0.45 1st Cohens d effect size: −1.0 2nd cluster *p*-value: 0.0116 2nd electrode: A31 2nd peak time: 2.8809 2nd r correlation effect size: −0.40 2nd Cohens d effect size: −0.88

**Table 4:** MUA results for the discomfort-by-decrease through the onsets with orthogonalized regressor. Only results for clusters containing significant effects (FWE-corrected) or borderline effects smaller than a *p*-value of 0.1 are shown, both positive and negative tails.

Effect	Tail (+1)	Tail (−1)
Discomfort by decrease 0.5–3.0 s	No significant Cluster	1st cluster *p*-value: 0.0004 1st electrode: B16 1st peak time: 2.8037 1st r correlation effect size: −0.53 1st Cohens d effect size: −1.24

**Table 5:** MUA results for the discomfort-by-increase (through the partitions) by decrease (through the onsets). Only results for analysis windows containing significant effects (FWE-corrected) or borderline effects (*p* < 0.1) are shown, both positive and negative tails.

Effect	Tail (+1)	Tail (−1)
Discomfort by increase in partitions by decrease in onsets 0.5–3.0 s	No significant Cluster	1st cluster *p*-value: 0.0686 1st electrode: B5 1st peak time: 1.7578 1st r correlation effect size: −0.28 1st Cohens d effect size: −0.58

## Data Availability

All code used to generate the analysis, figures, and results is available on GitHub at https://github.com/tomjefferis/FieldTrip-Pattern-Glare, the data is available upon request.
